# Theoretical and Experimental Investigations of Tool Tip Vibration in Single Point Diamond Turning of Titanium Alloys

**DOI:** 10.3390/mi10040231

**Published:** 2019-03-31

**Authors:** Wai Sze Yip, Suet To

**Affiliations:** State Key Laboratory in Ultra-precision Machining Technology, Department of Industrial and Systems Engineering, The Hong Kong Polytechnic University, Hung Hom, Kowloon, Hong Kong SAR, China; 13903620r@connect.polyu.hk

**Keywords:** material swelling, titanium alloys, single point diamond turning, tool tip vibration

## Abstract

The material swelling effect in single point diamond turning (SPDT) causes ragged materials on a machined surface which slows down the movements of tool tip vibration, and acts as a simple impacted pendulum system with a damping effect and displays a single twin peak in fast Fourier transform (FFT). Due to the low elastic modulus and low thermal conductivity of titanium alloys, the material swelling effect of titanium alloys in SPDT is much more serious than that of traditional metals. For this reason, the tool tip vibration in SPDT of titanium alloys is expected to be different from previous reports. In this study, apart from the demonstration of the original single twin peak induced from the material swelling effect by the main cutting motion, we reported recently that there exists another twin peak induced by secondary material swelling arising from the movement of tool tip vibration in the SPDT of titanium alloys. The additional twin peak was located at the right side of the original twin peak in FFT, displaying two twin peaks in the frequency domain of cutting force and suggesting the existence of another tool tip vibration system with a new damping factor in the SPDT of titanium alloys. Combining the effects of primary and secondary material swelling, the new dynamic model with the modified damping factor of tool tip vibration system are developed, which surface roughness of the machined titanium alloys in SPDT was predicted in higher accuracy by using the new model. The FFT of cutting force, surface roughness, and surface profile were provided in this article for the experimental validations.

## 1. Introduction

Ti–6Al–4V titanium alloys are widely applied into aircraft and medical industries due to their excellent physical properties such as high mechanical strength, light-weight, and good corrosion resistance. However, titanium alloys are treated as difficult to cut materials because of their low machinability. They have low thermal conductivity [1,2,3] and the high cutting heat is generated during machining processes, and the heat could not be dissipated from the cutting zone efficiently. The high amount of heat energy is trapped at the tool/work-piece interface, and as a result, cutting tools wear off rapidly because of high cutting temperature, noteworthy, adhesive tool wear occurs [4,5,6].

Micro machining is one of the manufacturing technologies to fabricate micro parts with complex features and geometries. The topic of material swelling in micro cutting has attracted academic concerns due to its decisive effect on surface roughness in micro machining. Plastically deformed materials are processed with elastic recovery on the clearance face of tool edge to generate a freshly machined surface in micromachining processes. As the material swelling effect, induced by the ploughing effect, was dominant and pronounced in the cutting tests, the generated cutting forces were observed by the researchers [7] and the relationship between the ploughing effect and material swelling was proven. Shawky and Elbestawi [8] suggested the source of process damping influenced by elastic recovery should be included in the dynamic model of tool tip vibration. The material swelling effect exerts a considerable impact into the tool tip vibration in micro machining processes.

The surface topography of machined surface in single point diamond turning (SPDT) involves few machining factors such as depth of cut, feed-rate, and spindle speed. By combining all the above machining factors, tool marks are generated on final machined surfaces because of integral effects of material swelling, and plastic deformation [9,10]. When a diamond tool starts to cut the machined surface, the materials near the tool edge suffer from a plastic side flow because of an insertion of high pressure from the tool edge [11]. That side flow materials finally leave on the machined surface, which the volume of the materials is normally expanded in response to the effect of thermal expansion, and finally, it causes a ragged machined surface, the phenomenon is called the material swelling effect, which is a common drawback in the SPDT of low thermal conductivity materials. The level of material recovery is highly related to material properties of machined materials. Various levels of material recovery occur in the machining of different materials in SPDT. Titanium alloys hold the material property of low elastic modulus. The strain rate of titanium alloys in the plastic deformation process is normally greater than 103 s^−1^ when the flow stress dramatically increases in the machining process [12,13,14], the above characteristics of titanium alloys intensify the level of material swelling of titanium alloys in SPDT. Low elastic modulus and high level of material swelling generate a large volume of ragged materials on the machined surface, and the ragged materials act as major barriers for tool tip movements, inducing a process damping effect to the tool tip vibration at a tool/work-piece interface. Therefore, the degree of tool tip vibration in the SPDT of titanium alloys is much more serious than that of other traditional metals, implying the different amplitudes of twin peak in the frequency domain analysis [15,16,17].

As the requirements in ultra-precision machining is demanding, only a slight instability in a machining process would cause a deterioration of surface quality. Therefore, the tool tip vibration is one of the significant factors influencing the surface quality of machined surface in SPDT of titanium alloys. The tool tip vibration on the ragged surface induced by material swelling generates the particular frequency and thus the special patterns display in frequency domain analysis. Cheung and Lee [17] suggested that the material induced vibration caused by material swelling generated the tool vibration with comparatively low frequency peaks in fast Fourier transform (FFT). Hocheng and Hsieh [18] proposed that the tool vibration generated the high frequency vibration of cutting force; this investigation concluded that the cutting force with a high frequency vibration was possibly caused by the tool condition. In the later works, researchers identified the twin peak in FFT of cutting force [19,20,21], they suggested that an appearance of twin peak in a frequency domain was an important characteristic to distinguish the vibration source. Furthermore, researchers suggested cutting motions would produce changes in microstructural material properties, which were regarded as material swelling in SPDT [9,22], and they added the damping effect induced from the material swelling effect on modeling of the tool tip vibration. Wang et al. [23] provided the new points on an appearance of twin peaks in FFT, they suggested that plastically deformed materials induced from material swelling should be treated as a damping factor, which the ragged surfaces caused by material swelling added the damping effect on the tool tip vibration of the SPDT system.

In this study, the damping effect induced by difficult to cut materials on the tool tip vibration of diamond turning was investigated. The materials for micro-cutting tests in Wang et al. [23] were mainly soft materials, i.e., aluminum alloys and copper; for this study, the materials for diamond turning were titanium alloys, which are harder materials, lower thermal conductivity, and higher material swelling recovery rate than that of copper and aluminum alloys. In addition to the findings in Reference [23], this study focuses on the materials properties of titanium alloys and their consequent effects on the tool tip vibration of single diamond turning. The above contributes to demonstrating a guideline for an investigation of the tool tip vibration in precision machining of difficult to cut alloys with similar material properties to titanium alloys. Titanium alloys cause a different level of material swelling on the machined surface in comparison to other materials, the comparison of the level of material swelling of different materials and that of titanium alloys was made and shown in Table 1. As shown in Table 1, the percentages of recovered materials of copper and aluminum were only 2% and 1% respectively while that of titanium alloys was 36.67%, the material swelling effect in diamond cutting of copper and aluminum alloys was relatively lower than that of titanium alloys. Titanium alloys are low elastic modulus materials, even small exertion forces induced from the tool tip vibration enable the inducing of secondary material swelling (SMS), causing another damping process and another tool tip vibration system in a SPDT process. SMS induced from the tool tip movement causes additional material pile-up on the machined surface, which seriously worsens the surface quality and further disturbs the assigned tool path for a nanometric surface roughness formation. SMS places serious effects on surface topography of machined surface in SPDT. In this study, the FFTs of cutting force generated in SPDT of titanium alloys at different feed-rates were investigated in detail, we discovered that an additional twin peak displayed next to the original twin peak under SPDT at all ranges of feed-rate. The pattern of new twin peak in FFT was similar to the original twin peak generated by the tool tip vibration in the main cutting motion, suggesting the existence of another tool tip vibration system. 

## 2. Experimental Setup

Two-phase titanium alloys, Ti6Al4V(TC4), were processed for SPDT. Ti6Al4V has 6% aluminum, 4% beta phase stabilizer vanadium, 0.25% iron, 0.2% oxygen, and titanium for the remaining parts. Titanium alloys were in cylindrical shapes with a diameter 15 mm. The radius of diamond tools was 1.468 mm and the height of the tool was 10.172 mm, which means the distance between diamond tool tip and the bottom of the tool holder. The force sensor Kistler 9256C (Kistler Instrumente AG, Winterthur, Switzerland) was used to measure the cutting forces in three directions. The sensitivity of Kistler set in the cutting tests was 24.63 pC/N. The sample rate of capturing force data used in Kistler was 50 kHz. In this study, Software “Dynoware” was chosen as a tool to generate FFT of cutting force. Generally, raw cutting force data was collected during single point diamond turning, which the cutting force generated would be sensed by the Kistler dynamometer. These cutting force signals would then be transferred to the amplifier, which is a built-in component of Kistler dynamometer. The amplifier would intensify the cutting force signals and transform to data acquisition unit. After collecting cutting force data in time domain, the data was converted in the form with voltage unit. It is a suitable form to input in the data analysis of Dynoware as cutting force data is needed to input into the Simulink program in Dynoware. Finally, the cutting force data was converted into the frequency domain by Dynoware, and FFT analysis could be conducted afterward. Moore Nanotech 350FG (4-axis Ultra-precision machine—three linear axes and one rotary axis, Moore Nanotechnology Systems, Swanzey, NH, USA) was used for SPDT, it enables to generate symmetric and freeform optical surfaces and other high precision components. it employs of a PC based Computer Numerical Control (CNC) motion controller with the reachable programming resolution 0.01 nm. Vacuum chuck installed in the ultra-precision machine is used for holding the fixture or the work-piece directly by using the sucking force. The nozzle is employed for delivering lubricants into the work-piece during SPDT. 

**Figure 1:**
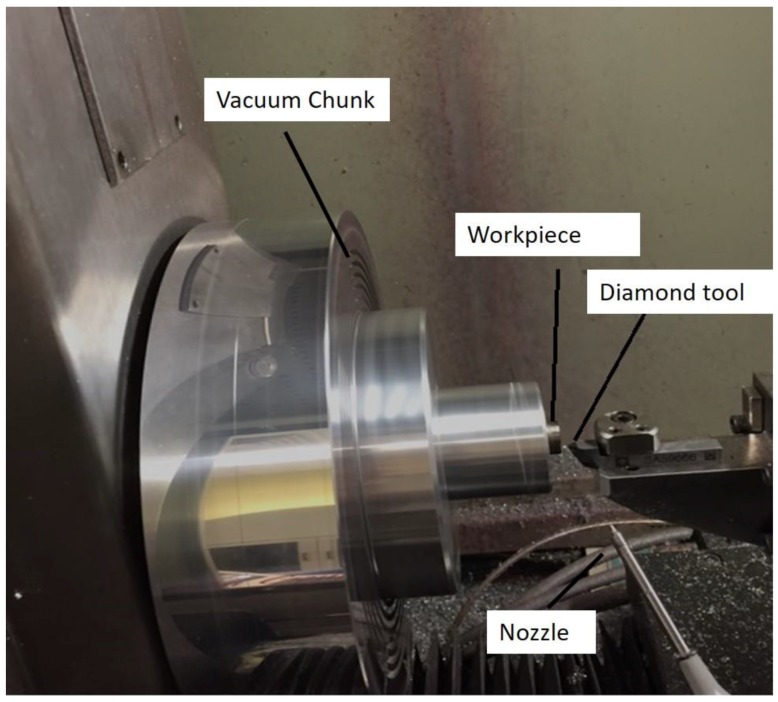
The experimental setup of single point diamond turning (SPDT) of titanium alloys.

Surface roughness was measured by Wyko NT8000 Optical Profiling System (WYKO Tire Technology, GreenBack, TN, USA) which is an optical profiler. Wyko NT8000 Optical Profiling System is capable of conducting a non-contact measurement of step heights, roughness, and surface topography of surfaces, and also, it can have gauge-capable measurement with sub-nanometer vertical resolution. Depth of cut and spindle speed were set as 4 μm and 1500 rpm, respectively, and were unchanged throughout the experiments. The feed-rate was set as variables, i.e., 2 mm/min, 4 mm/min, 6 mm/min, and 8 mm/min. All the experiments conducted in this study were in the lubricated condition. The feeding direction was parallel to the work-piece, the experiments in this study were face turning. The diamond tool condition was obtained after the cutting tests; it showed that the diamond tool had no crack formation, where the tool wear did not contribute an extra effect on the tool tip vibration in SPDT. The experimental setup is shown in Figure 1.

## 3. Theory

### 3.1. Hypothesis

For low elastic modulus materials especially titanium alloys, which its material swelling is relatively serious in comparison to other easy to cut materials in SPDT, the effects of their material swelling on the tool tip vibration in SPDT have rarely been studied previously. Titanium alloys have a high recovery rate and low elastic modulus, a small displacement of tool tip on the machined surface in the tool tip vibration induces another material swelling effect, causing secondary material swelling on the machined surface and further leading to an additional process damping effect in SPDT. Under this assumption, the process damping effect in SPDT of titanium alloys is logically presented to be various from Wang’s [23] model. The extremely small displacements parallel to the feed direction in the tool tip vibration could further generate swelled materials on top of the recovered surface, offering another tool tip vibration system with different vibration frequencies. Therefore, an additional twin peak with a smaller amplitude would appear in the FFT of cutting force in the SPDT of titanium alloys. Consequently, in SPDT of titanium alloys, the tool tip vibrates under the influences of two process damping factors, they are the recovered materials from primary material swelling (PMS) induced by the main cutting motion and the recovered materials from SMS induced by the tool tip vibration.

### 3.2. Theoretical Background

#### 3.2.1. Identification of Twin Peak from the Tool Tip Vibration Due to PMS Caused by the Main Cutting Motion

**Figure 2:**
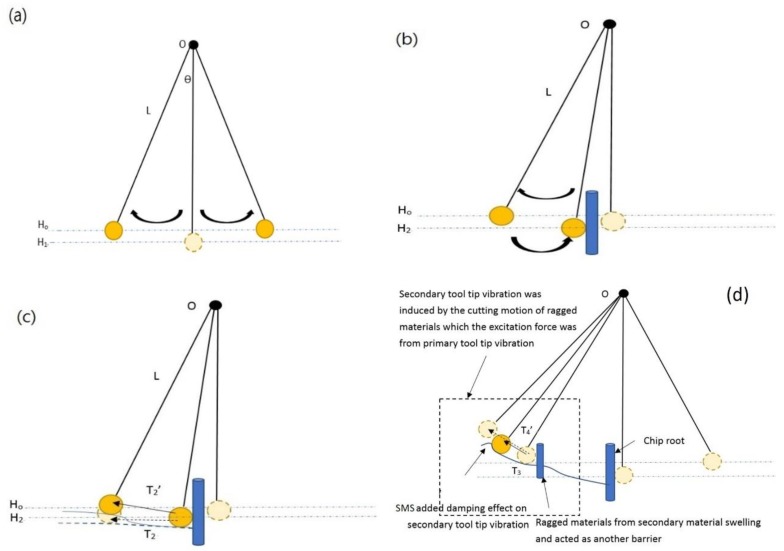
(**a**) The free pendulum system without an impact (**b**) the impact pendulum system without the damping effect and (**c**) the impact pendulum system with the damping effect. (**d**) The illustration graph of tool tip vibration induced by the ragged materials from secondary material swelling (SMS) and the corresponding damping effect.

The tool tip vibration with a damping factor in SPDT would be shown as a twin peak in FFT of cutting force [19,20,21]. The dynamic model of tool tip vibration with the material swelling effect is similar to an impacted pendulum system with a damping factor; its illustration graph is shown in Figure 2. 

Assumptions of simple harmonic motion of a pendulum system are made in this study, which are: The pendulum string is massless, the pendulum bob behaves as a mass point, the system is frictionless, and the impact is perfectly elastic with a small amplitude. The position function *h*_1_(*t*) of simple harmonic pendulum system without an impact is expressed as,

(1) $$h_{1}\left(t\right)=H_{1}\sin\left(\omega_{1}t+\varphi_{1}\right)$$

(2) $$\omega_{1}=\frac{2\pi }{T_{1}}=\frac{1}{\pi }\sqrt{\frac{g}{L_{1}}}$$

where *ω*_1_ and *φ*_1_ are the vibration frequency and the phase shift of simple harmonic pendulum without an impact respectively. *T*_1_ is the period of pendulum and *t* is the time which the pendulum is at the relative location in the position function. *g* is the acceleration of gravity, *θ* is the angle between the pivot point and the location of the pendulum at time *t*, the distance from the pivot point to the object is *L*_1_. As shown in Figure 2b, if a barrier is placed near the center of the pendulum system and the object gives an impact to it, the object direction would change with an unchanged velocity and magnitude. The position function of the pendulum *h*_2_(*t*) with an impact is now modified and termed as

(3) $$h_{2}\left(t\right)=H_{2}\sin\left(\omega_{2}t+\varphi_{2}\right)$$

(4) $$\omega_{2}=\frac{2\pi }{T_{2}}=\frac{1}{\pi }\sqrt{\frac{g}{L_{2}}}$$

where *ω*_2_ and *φ*_2_ are the vibration frequency and phase shift of the pendulum system with a presence of barrier respectively. Thus, the overall position function *h*(*t*) of the pendulum with a barrier in an excitation state is a summarization of the Equations (1) and (3)

(5) $$h\left(t\right)=h_{1}\left(t\right)+h_{2}\left(t\right)=H_{1}\sin\left(\omega_{1}t+\varphi_{1}\right)+H_{2}\sin\left(\omega_{2}t+\varphi_{2}\right)$$

Appling the scenario of tool tip vibration of SPDT into the above pendulum model, the chip root acted as a barrier in the pendulum system. The tool tip acted as a pendulum and the ragged materials from material swelling acted as damping objects in the pendulum impact mode. The diamond tool tip would be excited to vibrate when it impacted to the chip root in a material removal process. Therefore, the position function of the tool tip vibration induced by the impact between the tool tip and the chip root can be expressed as Equation (5). As shown in Figure 2c, due to the ragged surface generated in PMS, time *T*_2_′, which is denoted as the distance of tool tip backward movement in the vibration motion, is lengthened because of the recovered material surface. Therefore, the vibration frequency with the damping effect *ω*_2_′ in the tool tip vibration is smaller than *ω*_2_. In the meantime, *T*_2_′ > *T*_2_. The position function *h*′(*t*) in the impacted pendulum system with the damped factor is expressed as below

(6) $$h^{\prime }\left(t\right)=h_{1}\left(t\right)+{h_{2}}^{\prime }\left(t\right)=H_{1}\sin\left(\omega_{1}t+\varphi_{1}\right)+{H_{2}}^{\prime }\sin\left(\omega_{2}^{\prime }t+{\varphi_{2}}^{\prime }\right)$$

#### 3.2.2. Another Vibration System Induced from SMS Caused by the Tool Tip Vibration

Previous studies were focused on modeling the effect of material swelling on the surface generation and the tool tip vibration. Wang et al. [21,23] provided the experimental findings for the development of physical model of the correlation between characteristic twin peaks in frequency domain and the tool tip vibration in a micro cutting process. However, the effect of material swelling on various materials has been lacking. Due to the low elastic modulus of titanium alloys, it is believed that the small displacement in the tool tip vibration on the machined surface in SPDT of titanium alloys is able to induce SMS on the machined surface and creates another tool tip vibration system with a new process damping effect to the tool tip vibration.

**Figure 3:**
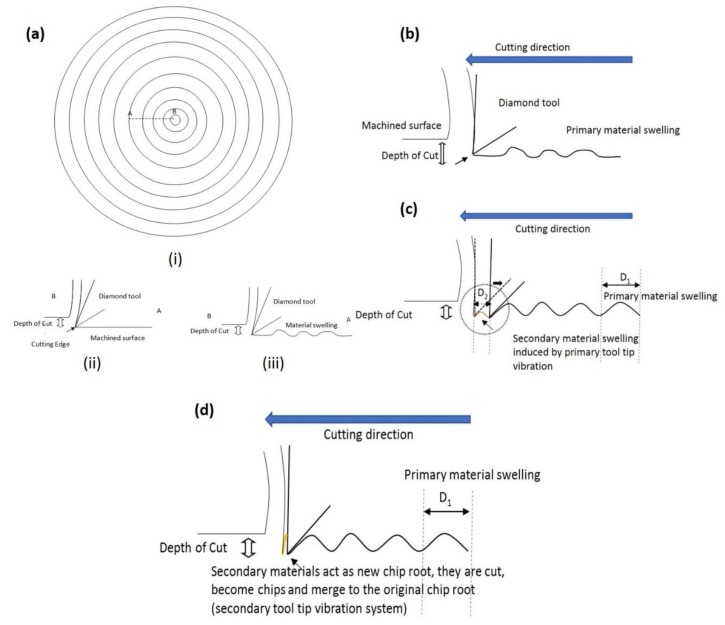
(**a**) The graphical illustration for material swelling generated in SPDT. (**b**) Primary material swelling (PMS) generated from the main turning motion. (**c**) SMS generated from the tool tip vibration. (**d**) The tool cuts the ragged materials of SMS and another tool tip vibration is generated.

The graphical illustration for material swelling generated in SPDT is shown in Figure 3a. In SPDT, when the cutting edge moves along the machined surface during a cutting motion, extremely small feed-rate involved in SPDT contributes to a nano-surface generation. Ideally, the diamond tool edge is small enough to generate tool marks with a narrow distance between every consequent tool mark, providing flatness surface. However, when the materials are recovered from the solidification process, they are expanded, and their volumes are increased on the machined surface. The final machined surface with recovered materials is wavy and ragged, they cause that the machined surface highly deviates from the ideal machined surface in SPDT. On the other hand, PMS is regarded as an elastic recovery from the plastic material deformation behind the cutting edge under the main turning process as shown in Figure 3b. Due to low elastic modulus and high sustainability of work hardening of titanium alloys, the machined surface of titanium alloys is easy to recover and swell. Therefore, it is believed that SMS is generated in front of tool edge after the backward movement in the tool tip vibration, which is shown in the dotted circle in Figure 3c. The ragged materials induced from SMS are in front of the cutting edge and they act as a second barrier. The ragged materials of SMS are treated as a new “chip root” and removed by the tool edge under the consequent vibration motion of the tool as shown in Figure 3d. Therefore, the ragged materials from SMS become the new process-damping factor in the tool tip vibration. Another vibration system due to the removal of ragged materials of SMS is generated, the tool tip is excited to vibrate with the new process-damping factor generated from SMS.

The impact pendulum system with the total damping effect combining with PMS and SMS is modified and shown in Figure 2d. Here, based on the impact pendulum model with the damping factor induced by PMS, the additional barrier on the impacted pendulum system standing for the ragged materials induced by SMS is added to the new model. *ω*_3_ was the new excited vibration frequency of the tool tip vibration induced from SMS without the damping factor, and *ω*_4_′ is the excited vibration frequency of tool tip vibration generated by passing through the ragged materials from SMS. As the ragged surface induced from SMS, time for the forward movement of tool tip vibration is lengthened, therefore, time period *T*_3_ is smaller than *T*_4_′, and *ω*_3_ is larger than *ω*_4_′. As a result, another twin peak representing the tool tip vibration induced from SMS appears in FFT; the peak of *ω*_3_ was at the right of the twin peak while *ω*_4_′ was at the left of the twin peak. On the other hand, as the excitation force of tool tip vibration in SMS is from the force of tool tip vibration in PMS, therefore, the amplitude of the twin peak for the tool tip vibration in SMS is smaller than that of in PMS. Consequently, the volume of ragged materials from PMS is larger than that of SMS, as shown in Figure 3c. The width of ragged materials arising from PMS *D*_1_ is larger than the width of ragged material arising from SMS *D*_2_. The above provides an additional information for identifying the locations of two twin peaks induced by the two tool tip vibration systems in PMS and SMS. 

For the additional tool tip vibration system induced from SMS, the effect of tool tip vibration induced by SMS should be added into the impacted pendulum system for representing the existence of two vibration systems in the SPDT of titanium alloys. Therefore, the position function *h*″(*t*) for the tool tip vibration in the SPDT of titanium alloys is:

(7) $$\begin{array}{cc} h^{\prime \prime }\left(t\right) & =h_{1}\left(t\right)+h_{2}^{\prime }+h_{3}\left(t\right)+h_{4}^{\prime }\\ & =H_{1}\sin\left(\omega_{1}t+\varphi_{1}\right)+{H_{2}}^{\prime }\sin\left({\omega_{2}}^{\prime }t+{\varphi_{2}}^{\prime }\right)\\ & \;+H_{3}\sin\left(\omega_{3}t+\varphi_{3}\right)+{H_{4}}^{\prime }\sin\left({\omega_{4}}^{\prime }t+{\varphi_{4}}^{\prime }\right) \end{array}$$

By adding the new concept of the damping factor induced by SMS, the equation of the tool motion in SPDT is developed. *C* is total damping coefficient of SPDT of titanium alloys, it includes two tool tip vibration systems induced by PMS and SMS, and the internal damping relation to turning system, where *C* is expressed as:

(8) $$C=C_{int}+C_{pms}+C_{sms}$$

where *C_int_* is the internal damping coefficient of SPDT system, *C_pms_* and *C_sms_* are the damping coefficient induced from PMS and SMS respectively. Based on the principle of balance force from Newton’s second law, the acting force of damped harmonic oscillation system is expressed as:

(9) $$F_{act}-Kx-c\frac{dx}{dt}=M\frac{d^{2}x}{dt^{2}}$$

where *M* and *K* are mass and stiffness of the diamond tool and chip roots related to the SPDT system respectively, *F_act_* and *x* are the external force acting on the tool and moving distance of the tool, respectively.

## 4. Experimental Validations

### 4.1. Two Individual Twin Peaks in FFT of Cutting Force of Titanium Alloys in SPDT

Under the assumption of existence of SMS, there would be another twin peak with the higher frequency in FFT of cutting force, forming as two twin peaks. The natural frequency of original turning motion in SPDT is determined as [23]:

(10) $$\frac{2\pi R\times spindle\;speed/60}{d}=\frac{2\pi \times 7.5\times 1500\mathrm{rpm}/60}{0.064}\;\sim \;18.4\mathrm{KHz}$$

18.4 kHz was determined by the surface waviness in the machined surface profile as shown in Equation (8) using the value of spindle speed, average separation distance *d* of tool marks at single diamond cut and size of the work-piece. On the other hand, refer to information from the literature review, Wang et al. [23] demonstrated the experimental results of tool tip vibration of SPDT of soft materials—copper and aluminum alloys, the excited natural frequency of tool tip vibration induced by the micro-cutting process of these two soft metals was about 14 kHz. In this proposed study, the materials for SPDT was titanium alloys, which are harder and higher strength materials than copper and aluminum alloys. Therefore, the excited natural frequency of tool tip vibration induced by SPDT of titanium alloys is higher than that of copper and aluminum alloys. The peaks locating below the frequency of P2 are not considered in detail in this study, as these low-frequency thrust forces do not directly contribute to the relative tool-work displacement, according to previous studies [24]. Only the cutting forces at the relatively high frequency (above 12 kHz) should be included in investigations of the mechanism of surface topography, and dynamic modeling of SPDT process in this study.

**Figure 4:**
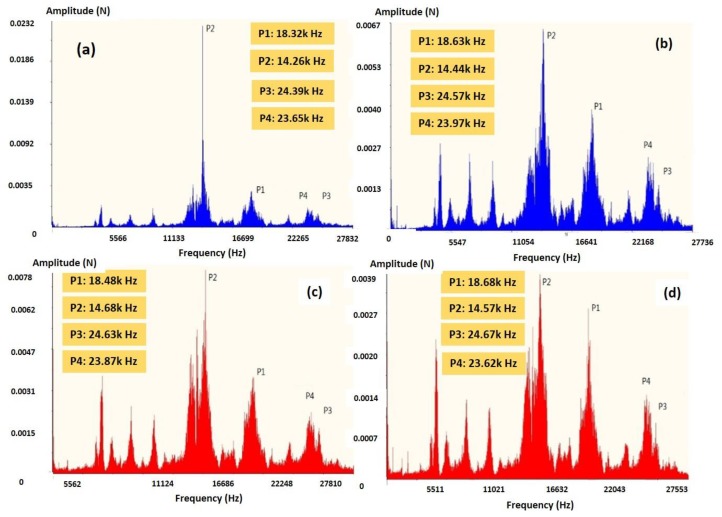
Fast Fourier transforms (FFTs) of SPDT of titanium alloys under feede-rate (**a**) 2 mm/min, (**b**) 4 mm/min, (**c**) 6 mm/min, and (**d**) 8 mm/min.

The characteristic twin peak, which denoted as the twin peak in FFT generated by the main cutting motion and PMS, was induced from the resonance frequency in SPDT and the frequency related to the impact between tool tip, chip root and the recovered materials from PMS. According to Figure 4, the peak P1 within the particular twin peak was appeared around 18.4 kHz in FFT of all ranges of feed-rate, which matched the natural frequency of turning system calculated above. Therefore, the twin peaks (P1 and P2) could be identified as the excited vibration frequency of the tool tip vibration with the damping effect generated in PMS. Recalling the above-mentioned, a small displacement in the tool tip vibration is expected to induce SMS on the machined surface. Therefore, another twin peak with a similar shape as the characteristic twin peak displays in FFT. Here, P4 and P3 peaks acted as another twin peak, which are positioned at the right side of the characteristic twin peak P1 and P2. P3 and P4 are identified as the twin peak representing the vibration system excited by the tool tip vibration in SMS, which have higher frequency and the smaller amplitudes than that of the tool tip vibration induced by PMS. 

**Figure 5:**
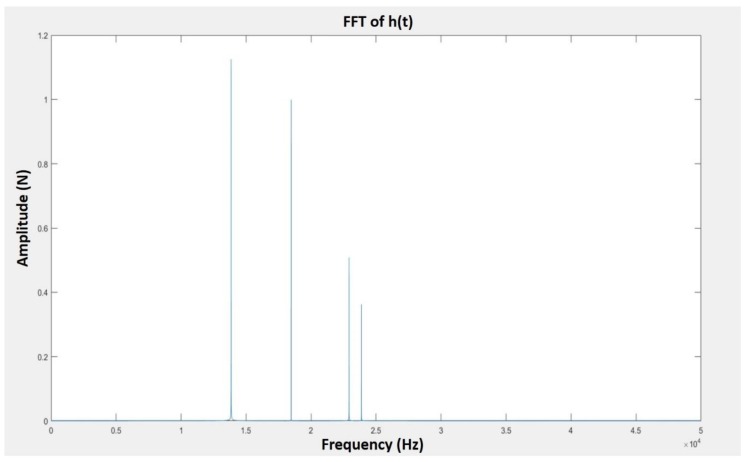
The FFT of the proposed impact pendulum model with the two vibration systems.

Equation (7) is transformed to the FFT in order to check if the corresponding patterns in the FFTs match the experimental results. Equation (7) is simplified according to the mathematic transformation of FFT, as shown in Figure 5 which *ω*_1_ = 116,000, *ω*_2_ = 87,000, *ω*_3_ = 150,000, *ω*_4_ = 144,000, *H*_1_ = 1, *H*_2_ = 1.7, *H*_3_ = 0.4, *H*_4_ = 0.6, and *t* = 0–50,000 s. According to Figure 5, the FFT pattern was matched to the experimental results as shown in Figure 4, which displayed two sets of twin peaks. The above results of FFT graph provide an experimental validation of the hypothesis stated in the above section and validated the modified model and the differential equation mentioned in the hypothesis.

### 4.2. Experimental Validations of Surface Roughness Influenced by PMS and SMS

An increase in the vibration force causes an increase of the amplitude of each peak in FFT, and simultaneously, the reinforcement of the vibration force would increase surface roughness. Therefore, the amplitude ratio of twin peak would reflect the trend of surface roughness generated in SPDT directly. The Characteristic Peak Ratio (*CPR*) was defined as the indicator for predicting surface roughness of machined surface in SPDT [23]. The relative amplitude of two peaks within the twin peak is described by *CPR*, which is expressed as

(11) $$CPR=\frac{A_{P1}}{A_{P2}}$$

where *A_P_*_1_ and *A_P_*_2_ are the amplitudes of *P*1 and *P*2, respectively. In this study, one more twin peak was found in SPDT of titanium alloys because of the process damping effect induced by SMS, which also contributes to influencing surface roughness in a surface generation. Therefore, the *CPR* should be modified to:

(12) $$CPR^{\prime }=n\frac{A_{P1}}{A_{P2}}+\left(1-n\right)\;\frac{A_{P3}}{A_{P4}}$$

where *CPR*′ is the modified indicator for predicting surface roughness in SPDT of titanium alloys with adding the effects of both PMS and SMS. As the influences of the two tool tip vibration systems on the surface generation are proportioned, therefore, the contribution percentage of the influences of PMS is added in Equation (12) and denoted as the weighting factor *n*, where *n* is smaller or equal to 1. For *n* = 1, the influence of damping effect in SMS does not contribute to surface roughness, where only the damping effect of PMS contributes to the surface generation. In this case, $\frac{A_{P3}}{A_{P4}}$ will be equal to zero and *CPR*′ = *CPR*, which is equal to the Wang model [23].

**Figure 6:**
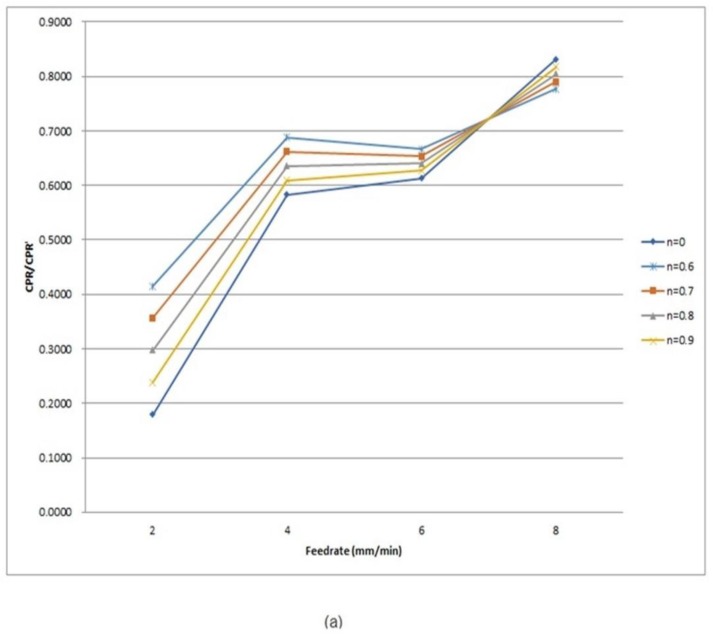

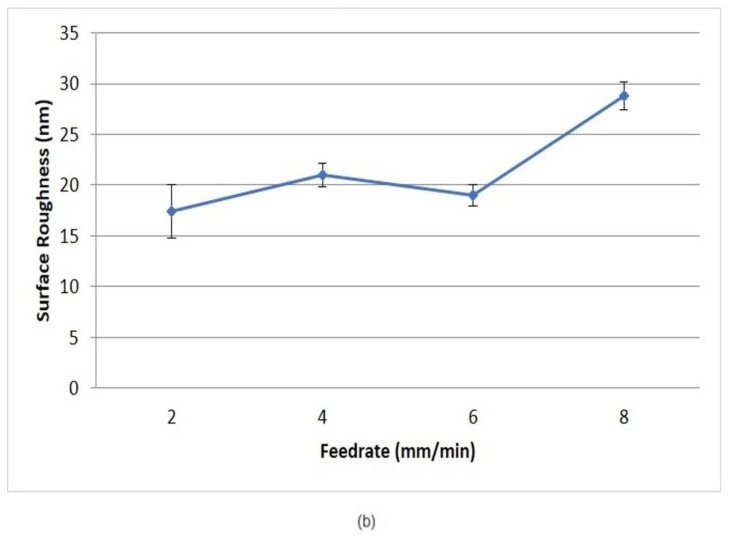
The graph of (**a**) *CPR* (Characteristic Peak Ratio)′ vs. feed-rate and (**b**) surface roughness vs. feed-rate.

The amplitude ratios of P2/P1 and P4/P3 generated in different feed-rate are shown in Table 2. The graph of *CPR*′ vs. feed-rate is shown in Figure 6a and surface roughness vs. feed-rate is shown in Figure 6b. The increasing/decreasing trends of lines in Figure 6a,b is compared with each other in order to show the consistency of the trend of *CPR*′ with surface roughness at different feed-rates. Comparing between Figure 6a,b, the decrease of surface roughness from feed-rate 4–6 mm/min in Figure 6b could not be presented by the curve of *CPR*′ at *n* = 1 in Figure 6a, also, the dramatic increase of surface roughness from feed-rate 2–4 mm/min at *CPR*′ at *n* = 1 in Figure 6a was not shown in Figure 6b too, therefore, the curve of *CPR*′ at *n* = 1 was not matched to surface roughness obtained from the experimental results. It makes the conclusion that the previous model of tool tip vibration is not valid in the case of SPDT of titanium alloys. On the other hand, the curve of *CPR*′ at *n* = 0.7 was the most fitted to the curve in Figure 6b. PMS is the dominant factor over SMS in the surface generation of SPDT of titanium alloys as the best fit curve at Figure 6a is *n* = 0.7, which is larger than 0.5. One of the noticeable points from Figure 6, surface roughness dropped at a feed-rate of 6 mm/min before increasing again at 8 mm/min. The possible reason for this phenomenon is that a higher feed-rate caused a stronger thermal softening effect on the cutting zone, which resulted in a decrease in cutting force, and consequently, surface roughness decreased at feedrate 6 mm/min before increasing again. The same experimental results were demonstrated in a similar study about the machining of titanium alloys [25].

### 4.3. Experimental Validation of Cutting Profile Generated by PMS and SMS

**Figure 7:**
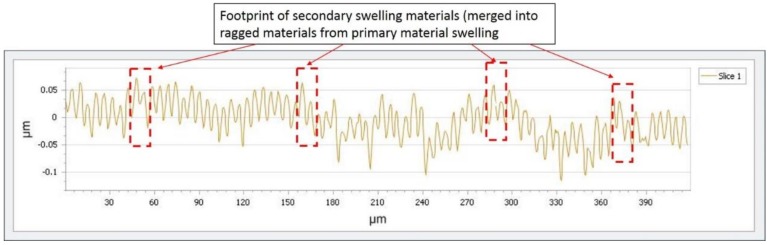
The surface profile of machined surface.

According to the hypothesis stated in this study, the materials induced by SMS are ragged and would be merged into the materials generated from the main cutting motion. In order to provide the experimental validation of the hypothesis, a cross section of cutting surface was captured by Wyko NT8000 Optical Profiling (WYKO Tire Technology) and is shown in Figure 7, which the cutting condition for this surface profile was the depth of cut 4 μm, spindle speed 1500 rpm and feed-rate 8 mm/min. As the volume of ragged materials from PMS is larger than that of SMS, therefore, as shown in Figure 3b,c, the width of ragged materials *D*_1_ arising from PMS are larger than the width of ragged material D_2_ arising from SMS, which would give out the tool mark with large and small widths simultaneously on one machined sample. According to the dotted squares in Figure 7, there were materials with shorter width and were merged into the cutting materials generated by the main cutting motion, which were possibility footprint of PMS and SMS on the machined surface. Like the previous section, the weighting factor “*n*” was determined as 0.7 for the best fit curve, therefore, the height of swelling material from SMS would be believed as approximately 70% of the height of swelling material from PMS. Therefore, the dotted profile was believed as the footprint of the ragged material of SMS. And this work is preliminary works for the tool tip vibration of titanium alloys in SPDT, which may provide elementary information for the further research with a similar topic. The accurate determination of the footprint of SMS would need to be further investigated in future research.

## 5. Conclusions

In this study, we focus on the particular material properties of titanium alloys, which are low elastic modulus and high material recovery rate, to investigate the material swelling effect on their machined surface in SPDT. Titanium alloys cause a different level of material swelling on the machined surface in comparison to other materials, introducing another tool tip vibration system induced by SMS and the new process damping factor in SPDT. In this study, we reported that there exists of the two tool tip vibration systems in SPDT of titanium alloys which were generated by PMS and SMS, and they were confirmed by the experimental validations. Under the consideration of two tool tip vibration systems, the new process damping factor was defined, and it was used to formulate the new total system damping factor, providing better accuracy to predict surface roughness of titanium alloys in SPDT.

## Figures and Tables

**Table 1:** The comparison of the level of material swelling of different materials and titanium alloys.

Materials	Assigned Cutting Depth (μm)	Average Cutting Depth of Machined Surface (μm)	Percentage of Recovered Material Volume (%)
Copper [1]	5	4.9	2
Aluminum [1]	5	4.95	1
Titanium alloys [2]	3	1.9	36.67

**Table 2:** The amplitudes of P1–P4 in the two twin peaks, the ratio of amplitude of P2/P1 and P4/P3 and surface roughness generated in different feed-rate.

Feedrate (mm/min)	Amplitude (N)				Amplitude Ratio		Surface Roughness (nm)
	P1	P2	P3	P4	P1/P2	P3/P4	
8	0.00278	0.00334	0.00091	0.00131	0.8315	0.6956	28.8
6	0.00360	0.00586	0.00158	0.00212	0.6134	0.7454	19
4	0.00371	0.00636	0.00162	0.00192	0.5825	0.8452	21
2	0.00402	0.02245	0.00168	0.00219	0.1792	0.7695	17.4
